# Novel inflammatory biomarkers in thyroid eye disease

**DOI:** 10.1530/EJE-22-0247

**Published:** 2022-06-08

**Authors:** Hans Olav Ueland, Grethe Åstrøm Ueland, Kristian Løvås, Lars Ertesvåg Breivk, Alexander Stanley Thrane, Ann-Elin Meling Stokland, Eyvind Rødahl, Eystein Sverre Husebye

**Affiliations:** 1 Department of Ophthalmology, Haukeland University Hospital, Bergen, Norway; 2 Department of Medicine, Haukeland University Hospital, Bergen, Norway; 3 Department of Clinical Science and K.G. Jebsen Center for Autoimmune Diseases, University of Bergen, Bergen, Norway; 4 Volvat Medical Center, Bergen, Norway; 5 Department of Medicine, Stavanger University Hospital, Stavanger, Norway; 6 Department of Clinical Medicine, University of Bergen, Bergen, Norway

## Abstract

**Purpose:**

The aim of this study is to identify biochemical inflammatory markers predicting the presence or risk of developing thyroid eye disease (TED) in patients with Graves’ disease (GD).

**Methods:**

Patients with GD (*n* = 100, 77 females) were included from the National Norwegian Registry of Organ-Specific Diseases. Serum samples were analysed for 92 different inflammatory biomarkers using the proximity extension assay. Biomarker levels were compared between groups of patients with and without TED and healthy subjects (HS) (*n* = 120).

**Results:**

TED was found in 36 of 100 GD patients. Significant (*P* < 0.05) differences in the levels of 52 inflammatory biomarkers were found when GD patients and HS were compared (42 elevated and 10 decreased). Out of the 42 elevated biomarkers, a significantly higher serum level of interleukin-6 (IL6) (*P* = 0.022) and macrophage colony-stimulating factor (CSF1) (*P* = 0.015) were found in patients with TED compared to patients without TED. Patients with severe TED also had significantly elevated levels of Fms-related tyrosine kinase 3 ligand (FLT3LG) (*P* = 0.009). Furthermore, fibroblast growth factor 21 (FGF21) was significantly increased (*P* = 0.008) in patients with GD who had no signs of TED at baseline but developed TED later.

**Conclusion:**

We demonstrate an immunologic fingerprint of GD, as serum levels of several inflammation-related proteins were elevated, while others were decreased. Distinctly increased levels of IL6, CSF1, FLT3LG, and FGF21 were observed in TED, suggesting that these inflammatory proteins could be important in the pathogenesis, and therefore potential new biomarkers for clinical use.

## Introduction

Thyroid eye disease (TED) is an inflammatory autoimmune disorder of the orbit affecting approximately 40% of patients with Graves’ disease (GD) (1). TED is five times more frequent in women than in men (reflecting the sex ratio in GD), with a peak incidence between 30 and 50 years of age (2). The condition consists of two phases; first, an active inflammatory phase, which gradually resolves into a second inactive fibrotic phase (3). Smoking, high levels of thyrotropin receptor antibody (TRAb), treatment with radioiodine, longstanding untreated hyperthyroidism, and hypothyroidism after initiation of treatment are known risk factors for the development and progression of TED (4, 5, 6, 7).

To diagnose early stages of TED can be difficult, especially if the eye symptoms precede hyperthyroidism, which occurs in 20–30% of the patients (8). Serum TRAb is a sensitive and specific biomarker for GD (9). Nearly all patients with TED have elevated TRAb levels, but not all patients with TRAb have orbitopathy. Further, osteopontin levels have been observed to correlate with the development of GD, but not with the presence of TED (10).

Raised levels of proinflammatory cytokines and chemokines, like interleukin (IL)1B, IL6 IL8, IL17, hepatocyte growth factor (HGF), interferon-gamma (INFG), and tumor necrosis factor-α (TNFα), have been observed in blood samples from TED patients compared with healthy subjects (HS) (11, 12, 13, 14). The levels of IL1B, IL6, and IL17 have been found to be different in the active compared to the fibrotic phase of the disease (15, 16). These studies are only observational and limited by small sample sizes, and none of the cytokines has been further validated as possible biomarkers of TED. A sensitive and specific biochemical marker for the presence of TED, or susceptibility to develop TED, is still lacking.

Multiplexing biomarker proteomics has been used to identify inflammatory markers in tear fluid in patients with TED (15, 17). Upregulation of several inflammatory proteins has been observed, with lysozyme C, lacritin, and zinc-α-2 glycoprotein 1 as the most interesting (18), but again only a few individuals with TED were analysed (19). Using the multiplex proximity extension assay, hundreds of biomarkers can be analysed in very small sample volumes. Here, we have applied this technique to analyse 92 different inflammatory biomarkers in relation to the presence of TED or the risk of developing TED.

## Patients and methods

### ROAS-registry and study population

The Norwegian Registry for Organ-Specific Autoimmune Disorders (ROAS) was established in 1996 and mainly includes patients with Addison’s disease and autoimmune polyendocrine syndromes. At the Department of Endocrinology, Haukeland University Hospital in the time period 2013–2020, 100 patients with GD were randomly recruited into ROAS. These patients were included by two dedicated doctors when logistics in a busy outpatient clinic allowed. At inclusion, the patients underwent a general clinical examination and blood sampling, after written informed consent for sharing biological material and personal health information in future research projects. The ROAS biobank has been approved by the Regional Committee for Medical and Health Research Ethics, Western Norway (IRB no. 00001872, ref. 2013/1504).

In the same period as the GD patients were included, serum samples were also collected from a group of 120 unmedicated HS without previous or ongoing thyroid dysfunction (Table 1). This group also signed an informed consent. HS were not matched one to one with the patients. By including 120 individuals, we were able to divide reference intervals into subgroups and correct for age, sex, smoking habits, and BMI for each biomarker. Serum samples from patients and controls were stored at −80°C until further analyses. The current study was approved by the Regional Committee for Medical and Health Research Ethics, Western Norway (ref. 2021/7624).

**Table 1:** Basic characteristics, blood levels, and treatment of hyperthyroidism at inclusion. Categorical data are given as *n* (%); continuous data are given as median (range).

Parameters	All	Patients with GD		Healthy subjects
		Without TED	With TED	
Basic characteristics				
Patients, *n*	100	64	36	120
Age, years (range)	42 (15–70)	43 (15–66)	41 (19–70)	40 (23–68)
Female, *n* (%)	77 (77)	48 (75)	29 (81)	66 (55)
Daily smoker, *n* (%)	22 (22)	8 (13)	14 (39)	1 (1)
Ex-smoker, *n* (%)	18 (18)	13 (20)	5 (14)	2 (2.7)
BMI, kg/m^2^ (range)	25 (17–49)	25.5 (17–49)	25 (35–18)	24 (16–34)
Systolic blood pressure, mmHg (range)	120 (90–192)	120 (90–192)	120 (100–170)	128 (109–170)
Heart frequency, beat/min (range)	72 (54–120)	72 (54–120)	73 (56–120)	
Inclusion within 2 months of hyperthyroidism, *n* (%)	59 (59)	38 (59)	21 (58)	
Duration of hyperthyroidism, months (range)	1 (0–519)	1 (0–519)	1 (0–365)	
Duration of TED, months (range)			9 (0–413)	
Anti-inflammatory treatment of TED*, *n* (%)			5 (14)	
Laboratory levels, median (range)				
s-free-Thyroxine, pmol/L	20 (7–81)	19 (7–81)	20 (13–75)	
s-Triiodothyronine, pmol/L	7.1 (1.3–31)	7.1 (1.3–31)	7.2 (3.6–31)	
s-TSH, mIU/L	0.01 (0.01–9.0)	0.01 (0.01–9.0)	0.03 (0.01–7.6)	
TRAb, IU/L	5.4 (1–40)	5.0 (1–21)	5.6 (1–40)	
TPO-ab >200 kIU/L, *n* (%)	37 (37)	27 (42)	10 (28)	
Type of treatment, *n* (%)				
Titration with carbimazole,	64 (64)	43 (67)	21 (33)	
Titration with prophylthiouracil	6 (6)	1 (1.6)	3 (8.3)	
Blocking with carbimazole	3 (3)	1 (1.6)	2 (5.6)	
Levothyroxine replacement	14 (14)	7 (10)	7 (19)	
No treatment of hyperthyroidism	11 (11)	10 (16)	1 (2.8)	
Unknown treatment of hyperthyroidism	2 (2)	0 (0)	2 (5.6)	

*Current or previous.

GD, Graves’ disease; TED, thyroid eye disease; TPOab, thyroid peroxidase antibody; TRAb, thyrotropin receptor antibody; TSH, thyroid-stimulating hormone.

### Clinical data

Clinical data at inclusion were obtained from the ROAS-registry, while follow-up data were retrieved from the hospital records. All patients were examined by an endocrinologist. Nineteen of the patients with TED including seven patients with Clinical Activity Score (CAS) (20) >3 were in addition examined by an ophthalmologist. Patients were categorized as having TED if they showed characteristic eye symptoms or findings at inclusion in ROAS or developed such symptoms or findings after inclusion. The following clinical characteristics of TED were registered: change in appearance, pain/pressure, diplopia, reduced vision, red eye, chemosis, periorbital swelling, eyelid retraction, strabismus, and exophthalmos. Severity of TED was classified according to the European Group of Graves’ Orbitopathy (EUGOGO)’s classification (3). Inflammatory activity was estimated using CAS. Active TED was defined as a CAS of three out of seven or higher.

### Laboratory data

Relevant laboratory data were obtained from ROAS and hospital records. Serum levels of free-thyroxine (fT4), thyroid-stimulating hormone (TSH), triiodothyronine (T3), thyroid peroxidase antibody (TPO-ab), and TRAb were all measured using electrochemiluminescence immunoassay (Roche Cobas®, Mannheim, Germany). Between-day coefficient of variation (CV) for fT4, TSH, T3, and TPO-ab were 5, 5, 7, and 8%, respectively. For TRAb, CV was between 5% at 16.0 IU/L and 13% at 1.7 IU/L. All the assays were sandwich methods, except for TSH, which was a competitive assay. Cut-off level for positive TRAb was 1.75 IU/L and was 200 kIU/L for TPO-ab. Normal range in adults for fT4 was 9.5–22 pmol/L, for TSH 0.4–4.5 mU/L, and for T3 1.2–3.1 pmol/L.

### Analysis of inflammatory markers

A total of 220 serum samples were analysed by multiplex proximity extension assay for 92 different inflammation markers (Proseek Multiplex Inflammation I panel, Olink Bioscience, Uppsala, Sweden (Olink)) using 40 µL of serum. Olink’s inflammation panel includes a broad range of proteins associated with inflammatory diseases or related biological processes. Briefly, analysis was performed by use of oligonucleotide-labelled antibody probe pairs binding to their respective target protein. Then, a proximity-dependent DNA polymerization step was performed, resulting in a reported sequence that could be measured by quantitative PCR (21). Each assay was expressed as relative quantification between samples, by a unit named Normalized Protein eXpression (NPX). As NPX values are in log_2_-scale, a difference in one NPX equals a doubling in protein concentration. Further details regarding the limit of detection (LOD) and CV for the essential biomarkers are given in the Supplementary Table (see section on supplementary materials given at the end of this article).

### Statistical analysis

Descriptive statistics was used to describe the cohorts/subgroups. Student’s *t*-test or Mann–Whitney *U*-test was used as appropriate to detect differences in biomarkers between groups. When appropriate, Benjamin Hochberg procedure for adjustment of *P*-value was used to correct for possible false discovery rate due to multiple comparisons. NPX values below LOD were set as LOD. A normal range for each biomarker was defined based on the 2.5 and 97.5 percentiles in HS. Dixon’s criteria was used to define outliers (22). Harris and Boyd’s criteria were used to assess the need for dividing reference intervals into subgroups according to sex, age, and diurnal variation (23). For potential new biomarkers, the proportion of patients outside the defined normal range was calculated. Receiver operating characteristic (ROC) analyses were used to assess the diagnostic performance of the potential biomarkers. Spearman’s correlation (rho) was used to estimate the correlations between biomarkers and factors (age, sex, BMI, smoking status, and thyroid status) that possibly could affect the results. If necessary, results were corrected for confounding factors by linear regression. Multiple linear regression analyses were performed to detect associations.

## Results

### Study population

Of 100 patients (77 females) with GD, 29 had clinical characteristics of TED at inclusion, and another 7 developed TED within 72 months. A control group of 120 HS (66 females) was used for comparison. The GD patients’ median (range) age at inclusion was 41.5 (15–70) years. At inclusion, 40 patients were hyperthyroid, 1 was hypothyroid and 59 had thyroid hormone levels in the normal range. Fifty-nine patients were included within 2 months after the presentation of their hyperthyroidism. Clinical characteristics and laboratory data of patients and HS are presented in Table 1. Thirty patients had additional autoimmune disorders and two patients had more than one.

A general practitioner most often started treatment with carbimazole before referral. At the time of inclusion, 75 received treatment for their hyperthyroidism (Table 1). The patients’ follow-up data revealed a median (range) maximum fT4 level during the disease course of 38 (13-100) pmol/L and a median maximum TRAb level of 24 (4–380) IU/L. Median number of relapses of hyperthyroidism was 1 (1–4). At a median of 36 (6–240) months after the first episode of hyperthyroidism, 34 had received definitive treatment by either radioiodine (*n* = 18), or total thyroidectomy (*n* = 10), and 6 by a combination of both modalities. In nine patients, the indication for definitive treatment was TED. During radioiodine treatment, seven patients received prednisolone. After radioiodine treatment, three patients developed TED. One of these patients had received prednisolone according to EUGOGO’s guidelines (3).

**Figure 1:**
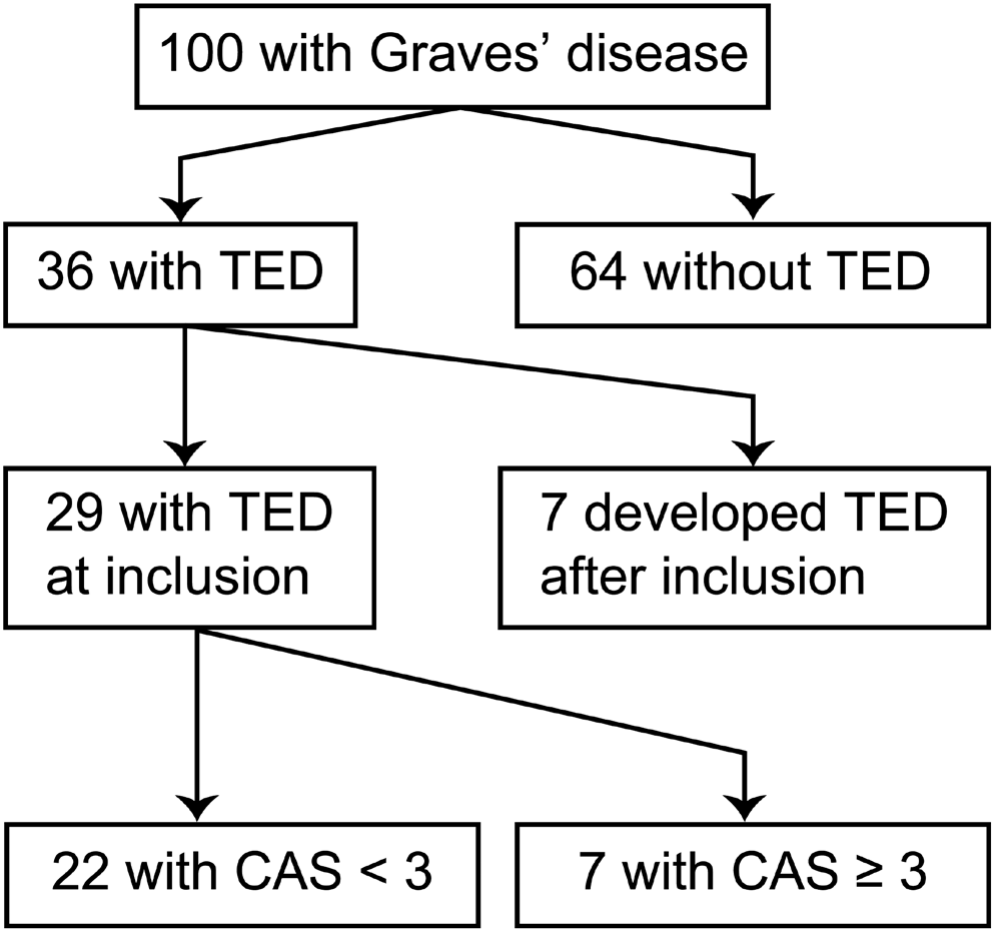
Patients with Graves’ disease included in the study classified according to TED. TED, thyroid eye disease; CAS, clinical activity score.

Among the 36 patients with TED, 29 had clinical signs and/or symptoms of TED at inclusion, with a CAS ≥3 in 7 patients. Median (range) time after diagnosis of Graves’ hyperthyroidism to development of TED was 0 (0–240) months. Seven patients developed TED after inclusion according to the hospital records (Fig. 1). Two of these developed TED within 6 months of inclusion. The severity of TED according to EUGOGO’s classification was mild in 27, moderate to severe in eight, and sight-threatening in one.

We found no significant difference in age and BMI between the patients with GD and HS, but there were significantly less females and smokers among HS (*P* < 0.05). The proportion of daily smokers were 22% in the GD group, 38.9% in the TED group, and 1% among HS. Further, the TED group had a lower proportion of patients with positive TPO-ab (>200 kIU/L), than the whole GD group. Otherwise, no significant differences in routine laboratory analyses were observed between the GD patients with and without TED.

### Biomarkers

**Figure 2:**
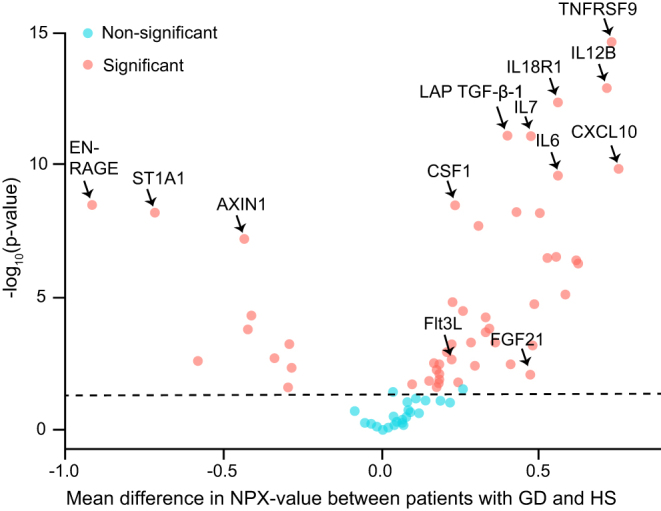
Volcano plot comparing 120 HS and 100 patients with GD, where 52 out of 92 inflammatory biomarkers show a statistically significant difference in median serum NPX levels between the two groups. Benjamin Hochberg procedure was applied to adjust P-values for statistical significance to control for false discovery rate due to multiple comparison. GD, Graves’ disease; HS, healthy subjects; NPX, Normalized Protein eXpression. A full colour version of this figure is available at https://doi.org/10.1530/EJE-22-0247.

For 73 out of 92 biomarkers, 90% of NPX values were above LOD. After correcting for multiple comparisons (adjusted *P*-values < 0.05), levels of 52 inflammatory biomarkers differed between patients with GD and HS (Fig. 2). Forty-two biomarkers were higher in the GD group while 10 were lower. Serum levels of 10 biomarkers correlated (r > 0.2, *P* < 0.05) with the level of fT4 and 12 with the level of TRAb (Supplementary Table).

Six biomarkers showed a highly significant increase (*P* < 1 × 10^−8^) between patients with GD and HS (Fig. 2), most significant for TNF receptor superfamily member 9 (TNFRSF9). By multiple linear regression analyses (dependent variable = biomarker), TNFRSF9 showed a moderate association with fT4 (*P* = 0.024, β = 0.233) and TRAb (*P* = 0.002, β = 0.314) and negative association with BMI (*P* = 0.013, β = −0.245). As a biomarker for GD, a sensitivity of 80% and specificity of 70% for TNFRSF9 were found by ROC-analysis using an optimal NPX cut-off level of 7.84 (Supplementary Fig. 1). We found that 39 out of 100 patients with GD had a serum level of TNFRSF9 above the 97.5 percentile for HS.

We found no difference in serum levels of the four most significantly suppressed biomarkers in patients treated with carbimazole (*n* = 67) and propylthiouracil (*n* = 6) compared to the remaining patients (*n* = 27).

**Figure 3:**
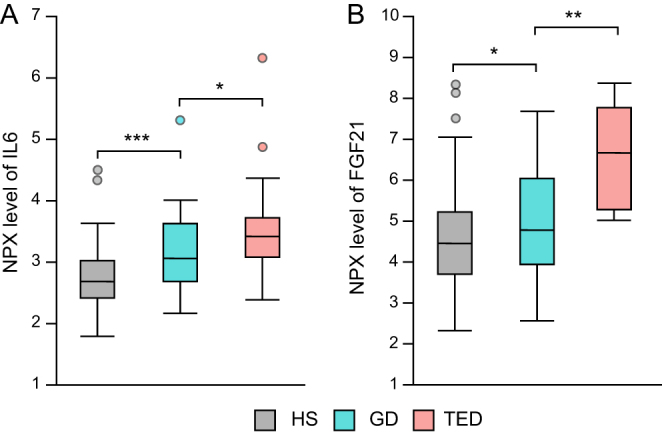
Boxplot for serum NPX level of (A) IL6 for HS, and GD with and without TED. (B) FGF21 for HS, and GD patients with and without TED. *P < 0.05. **P < 0.005. ***P < 0.0005. FGF-21, fibroblast growth factor21; GD, Graves’ disease; HS, healthy subjects; IL6, interleukin-6; NPX, Normalized Protein eXpression; TED, thyroid eye disease. A full colour version of this figure is available at https://doi.org/10.1530/EJE-22-0247.

Among the 42 biomarkers with an increased level of GD, without correcting for multiple comparisons, a difference in serum levels between the GD patients with and without TED was observed for IL6 (*P* = 0.022) and macrophage colony-stimulating factor 1 (CSF1) (*P* = 0.015). After correcting for smoking habits, the differences were still significant for IL6 (*P* = 0.03) and a trend was seen for CSF1 (*P* = 0.08). Compared to serum levels in HS, 29% of patients with TED had values above the 97.5 percentile for IL6 and 18% above the 97.5 percentile for CSF1. Figure 3A shows significantly higher serum levels of IL6 in GD patients without TED compared to HS (*P*< 0.0001), and in GD patients with TED compared to those without TED (*P* = 0.022). We found a weak correlation between serum level of TRAb and both IL6 (r = 0.256, *P* = 0.014) and CSF1 (r = 0.245, *P* = 0.019).

When comparing serum level of the 42 elevated biomarkers between those with serious TED (EUGOGO grades 1 and 2, *n* = 9) and mild TED (EUGOGO grade 3, *n* = 27), we found that Fms-related tyrosine kinase 3 ligand (FLT3LG) was significantly elevated (unadjusted and adjusted for smoking, *P*-values = 0.009). We found a weak association between FLT3LG and TRAb levels (*P* = 0.024, β = −0.254), but TRAb was not significantly different between patients with serious and mild TED.

### Predictors of developing TED

To identify a biomarker for patients at risk of developing TED, we examined the level of inflammatory markers in the seven patients who developed TED after inclusion. A difference in the level of fibroblast growth factor 21 (FGF21) (unadjusted *P* = 0.008) was observed between patients who developed TED and those who did not. After correcting for smoking habits, the difference in FGF21 was still significant (*P* = 0.003). Three out of seven who developed TED had a serum level of FGF21 above the 97.5 percentile for HS. Figure 3B shows significantly higher serum levels of FGF21 in GD patients without TED compared to HS (*P* = 0.016), and in GD patients who developed TED after inclusion compared to GD patients without TED (*P* = 0.003). FGF21 had a sensitivity of 89% and specificity of 53% (Supplementary Fig. 2) for the development of TED, estimated by ROC-analysis using an optimal NPX cut-off level of 5.10. In comparison, TRAb had a sensitivity of 71% and specificity of 51% for the development of TED, estimated by ROC-analysis using an optimal cut-off level of 4.99 IU/L. In HS, FGF21 in serum correlated with BMI (r = 0.182, *P* = 0.048), but not with age. We found no significant difference in BMI between patients who developed TED and those who did not.

## Discussion

We here demonstrate an elevation of two inflammatory biomarkers, IL6 and CSF1, in serum from GD patients with TED compared to patients without TED. In addition, FLT3LG was higher in patients with serious TED than in moderate TED. We also found elevated FGF21 levels in patients without signs of TED at baseline, but who developed TED later. The information can be useful to personalize treatment and follow-up of patients with GD.

In the orbital inflammatory process, IL6 is secreted by orbital fibroblasts. Further, IL6 has been observed to increase the expression of TSH-receptors on orbital fibroblasts and to stimulate plasma cells to antibody production (24, 25). Raised levels of IL6 have been found in tear fluid from active compared to inactive TED (26), and Molnar et al have described high serum levels of IL6 in TED (27). Inhibition of IL6 by tocilizumab has proven to be an effective therapeutic option in patients with steroid-resistant TED (28). Our finding of a systemic elevation of IL6 in patients with TED could support a broader use of the IL6 receptor antibody tocilizumab in the treatment of TED.

CSF1 is a hematopoietic growth factor which causes stem cells to differentiate into monocytes, macrophages, and dendritic cells (29). It binds to the CCR2-receptor and elicits a chemotactic signal in mononuclear cells to migrate to the site of inflammation. CSF1 is involved in the pathogenesis of different chronic inflammatory diseases including rheumatoid arthritis (RA) (30) and systemic lupus erythematosus (31). To our knowledge, elevated serum levels of CSF1 have not been described earlier in patients with TED.

FLT3LG is also a cytokine known to be involved in the mobilization and differentiation of hematopoietic stem cells and it has been linked to autoimmune disorders (32), but previously not to GD and TED. Migration of immature, bone marrow-derived fibrocytes to the inflamed orbital tissue in response to the release of cytokines is thought to be a key event in the pathogenesis of TED (33). Orbital fibrocytes have the capabilities to differentiate into myocytes and adipocytes. Preventing fibrocyte migration to the site of orbital inflammation is most likely beneficial in TED.

Identification of patients with GD who are at risk of developing TED is of utmost importance in clinical practice. It could reduce the risk of induction or exacerbation of TED by radioiodine treatment. Orbital volume expansion in TED is partly due to enhanced adipocytogenesis (34). FGF21 is known to stimulate glucose uptake in adipocytes via the induction of glucose transporter GLUT1/SLC2A1. We detected a higher serum level of FGF21 at inclusion in patients who later developed TED compared to those who did not. FGF21 has been reported to correlate with CAS in TED patients (35). This suggests that FGF21 could be involved in stimulating increased orbital glucose metabolism, making FGF21 a possible biomarker for the development of TED.

In our typical cohort of patients with GD, we observed a higher serum level of 42 different inflammatory biomarkers in patients with GD than in HS, while 10 markers showed lower levels. Previous studies have shown a rise in one or a few of these biomarkers in GD (36), but here we demonstrate a broad systemic immune response.

We also found elevated serum levels of biomarkers not previously reported in GD. TNFRSF9 is a TNF receptor, and together with its ligand 4-1BBL, it has been observed at sites of inflammation in RA (37). We found that TNFRSF9 had a moderate association with both fT4 and TRAb. TRAb is a highly specific and widely applied biomarker for GD. In the current study, we observed a sensitivity of 80% and specificity of 70% for TNFRSF9 as a biomarker for GD. This is inferior to earlier reports on TRAb (sensitivity 97.2%, specificity 99%) (9).

Inflammatory bowel diseases (IBD) is one of few autoimmune diseases studied by multiplex proximity extension assays (38). Compared to the inflammatory profile in patients with GD, a distinct difference is observed, as the most suppressed biomarkers in GD seem to be those with the largest increase in IBD. Thus, we have demonstrated the immunologic profile in GD, which is different from the profile of IBD.

There are some limitations to our study. The proximity extension immunoassays measure the relative levels of serum biomarkers. Our results could therefore not be compared to studies where traditional immunoassays have been used. To overcome this, we generated our own reference intervals for all biomarkers using 120 HS. For 73 out of 92 biomarkers 90% of assays were above LOD. In addition, only one outlier was detected in analyses of serum from HS. Together, this demonstrates the robustness of the method. The finding of associated autoimmunity in 30% of the patients could bias our findings.

By applying proteomics to a broad panel of 92 biomarkers, there is an increased likelihood of chance findings. We corrected this by applying the Benjamin Hochberg procedure to adjust the *P*-value for statistical significance when analysing differences in serum of inflammatory markers between patients and HS. To avoid excluding important observations, adjusted *P*-values were not used when differences between patients with and without TED were analysed. Therefore, these findings must be interpreted with caution.

In the present study, the difference in smoking habits across study groups could be a major confounder since smoking is a well-known risk factor for TED (39). We adjusted for smoking habits by linear regression and found that it did not affect our findings.

In conclusion, our study demonstrates a novel immunological fingerprint in GD, with particularly high levels of IL6, CSF1, FLT3LG, and FGF21 in TED, suggesting that these are important in the pathogenesis. Their measurement could prove valuable in order to personalise treatment, for example, with tocilizumab and to follow the treatment course. Finally, FGF21 could be a novel biomarker for assessing the risk of development of TED. The clinical use of these biomarkers should be tested further.

## Supplementary Material

Supplementary Figure 1. ROC-curve for Tumour necrosis factor receptor superfamily member 9 (TNFRSF9) as a biomarker for Graves` disease showing an area under the curve (AUC) of 0.82 (CI 0.76-0.88, p<0.05). The optimal cut-off level in our material for TNFRSF9 of 7.84 yielded a Sensitivity of 80% and specificity 70%.

Supplementary Figure 2. ROC-curve for Fibroblast growth factor-21(FGF-21) as a biomarker for development of thyroid eye disease in patients with Graves` disease showing an area under the curve (AUC) of 0.78 (CI 0.65-0.96, p<0.05). The optimal cut-off level in our material for FGF-21 of 4.99 yielded a sensitivity of 89% and specificity of 53%.

Supplementary Table. Distribution of 52 biomarkers with significantly different serum levels in patients with GD versus healthy subjects

## Declaration of interest

The authors declare that there is no conflict of interest that could be perceived as prejudicing the impartiality of the research reported.

## Funding

This work was supported by grants from the Norwegian Thyroid Association and Regional Health Authorities of Western Norway.
