# Supplementary figures and images for: Novel inflammatory biomarkers in thyroid eye disease
Source: Eur J Endocrinol. 2022 Jun 8;187(2):293–300. doi: 10.1530/EJE-22-0247 (PMC9723260; doi:10.1530/EJE-22-0247)

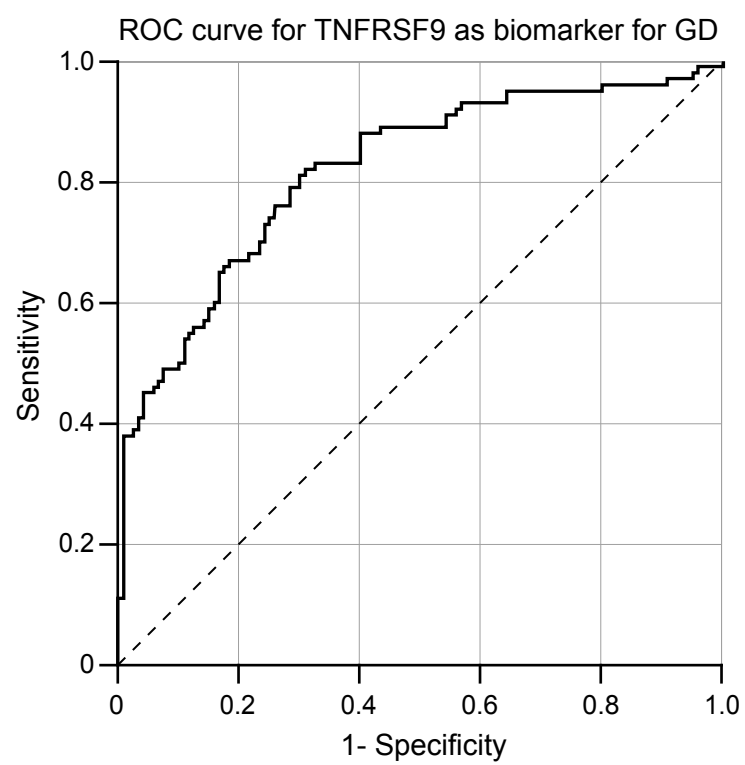

Supplement: Supplementary Figure 1. ROC-curve for Tumour necrosis factor receptor superfamily member 9 (TNFRSF9) as a biomarker for Graves` disease showing an area under the curve (AUC) of 0.82 (CI 0.76-0.88, p<0.05). The optimal cut-off level in our material for TNFRSF9 of 7.84 yielded a Sensitivity of 80% and [file supplementary_figure_1.pdf]

ROC curve for FGF21 as biomarker for development of TED

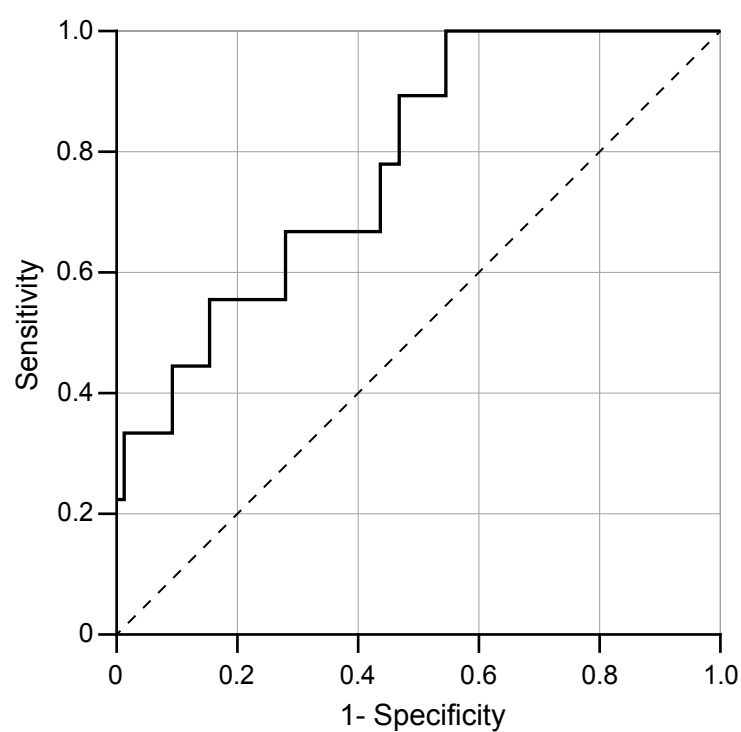

Supplement: Supplementary Figure 2. ROC-curve for Fibroblast growth factor-21(FGF-21) as a biomarker for development of thyroid eye disease in patients with Graves` disease showing an area under the curve (AUC) of 0.78 (CI 0.65-0.96, p<0.05). The optimal cut-off level in our material for FGF-21 of 4.99 yielded  [file supplementary_figure_2.pdf]
