# Supplementary material for: Novel inflammatory biomarkers in thyroid eye disease
Source: Eur J Endocrinol. 2022 Jun 8;187(2):293–300. doi: 10.1530/EJE-22-0247 (PMC9723260; doi:10.1530/EJE-22-0247)
Supplement: Supplementary Table. Distribution of 52 biomarkers with significantly different serum levels in patients with GD versus healthy subjects [file supplementary_table_1.pdf]

Supplementary Table. Distribution of 52 biomarkers with significantly different serum levels in patients with GD versus healthy subjects

	Patients with GD (n=100)	Healthy subjects (n=120)					
**Biomarker**	**Median (min-max)**	**Median**	**2.5- (min-max)**	**97.5-percentile percentile**	**LOD (90% CI)**	**CV**	**p-value[*] (90% CI)**
**Increased**							
Tumor necrosis factor receptor superfamily member 9[‡,§]	8.30 (6.44-10.09)	7.64 (6.74-9.31)	6.81 (6.75-6.90)	8.53 (8.34-9.28)	2.51	5%	0.0000
Interleukin-12 subunit beta[§]	7.70 (5.34-9.30)	7.05 (5.56-8.29)	5.85 (5.57-5.99)	8.13 (7.92 - 8.28)	0.51	6%	0.0000
Interleukin-18 receptor 1[‡]	9.53 (8.57-11.31)	9.07 (7.87-10.32)	8.17 (7.89-8.36)	10.11 (9.82-10.32)	2.37	5%	0.0000
Latencyassociated peptide transforming growth factor beta-1[‡,§]	8.87 (8.20-9.86)	8,53 (7.17-9.56)	7.74 (7.22-7.95)	9.34 (9.18-9.55)	1.80	7%	0.0000
Interleukin-7	4.26 (3.02-5.60)	3.38 (2.54-4.96)	2.76 (2.56-3.10)	4.60 (4.49-4.96)	1.70	6%	0.0000
C-X-C motif chemokine 10	10.46 (8.79-13.29	9.74 (7.92-12.45)	8.06 (7.97-8.44)	12.28 (11.18-12.45)	7.63	7%	0.0000
Interleukin-6	3.18 (2.16-6.33)	2.67 (2.12-4.49)	2.12(2.12-2.12)	3.62 (3.51-4.48)	2.12	6%	0.0000

Macrophage colonystimulating factor 1[§]	11.38 (10.79-13.02)	11.17 (10.5211.72)	10.60 (10.5310.67)	11.60 (11.53-11.72)	2.31	5%	0.0000
Hepatocyte growth factor	10.23 (9.05-11.56)	9.78 (8.36-10.76)	8.52 (8.36-8.85)	10.68 (10.50-10.76)	2.07	6%	0.0000
CLUB domaincontaining protein 1	3.68 (2.46-5.78)	3.27 (2.16-5.62)	2.28 (2.16-2.47)	5.10 (4.20-5.60)	0.90	6%	0.0000
Interleukin-10 receptor subunit beta[‡,§]	7.28 (6.41-8.33)	7.04 (5.17-7.76)	6.20 (5.23-6.37)	7.67 (7.58-7.76)	1.73	6%	0.0000
Interleukin-10[†,§]	4.82 (3.30-8.47)	4.37 (3.48 6.29)	3.57 (3.48-3.65)	5.66 (5.2-6.28)	2.63	7%	0.0000
Transforming growth factor alpha	5.76 (3.81-7.60)	5.11 (3.47-6.50)	3.65 (3.47-4.02)	6.29 (6.15.6.48)	1.85	6%	0.0000
C-X-C motif chemokine 9	8.00 (6.09-10.12)	7.25 (5.60-11.11)	5.91 (5.61-6.36)	9.88 (8.71-11.11)	2.17	6%	0.0000
Oncostatin-M	7.52 (4.81-10.51)	6.86 (4.77-8.42)	5.24 (4.79-5.72)	8.21 (8.13-8.41)	1.80	5%	0.0000
Interleukin 8	7.37 (5.43-14.10)	6.91 (4.85-9.10)	5.28 (4.29-5.71)	8.54 (8.17-9.06)	2.18	6%	0.0000
Interleukin-15 receptor subunit alpha[‡,§]	2.35 (1.68-3.78)	2.17 (1.37-2.98)	1.57 (1.38-1.74)	2.91 (2.69-2.97)	1.35	6%	0.0001
C-X-C motif chemokine 3[‡,§]	7.40 (5.84-13.78)	6.95 (5.49-8.59)	5.81 (5.50-6.06)	8.34 (7.99-8.59)	2.25	6%	0.0001
T-cell surface glycoprotein CD5	6.62 (5.37-7.72)	6.38 (5.21-7.37)	5.60 (5.21-5.72)	7.28 (7.05-7.37)	1.51	5%	0.0001
C-X-C motif chemokine 5	13.55 (11.62-14.40)	13.05 (10.7614.44)	11.51 (10.8112.08)	14.36 (14.10-14.43)	2.56	7%	0.0002
Interleukin-18	9.59 (7.91-11.21)	9.26 (7.9-10.67)	8.23 (7.91-8.45)	10.49 (10.16-10.66)	1.65	6%	0.0006

C-X-C motif chemokine 4	8.01 (6.12-9.36)	7.58 (6.22-9.32)	6.43 (6.22-6.60)	9.06 (8.47-9.31)	2.23	6%	0.0007
Monocyte chemotactic protein 3	3.10 (1.91-8.0)	2.80 (1.91-4.55)	1.92 (1.91-2.13)	4.40 (3.72-4.54)	1.91	7%	0.0016
Vascular endothelial growth factor A	12.45 (10.97-13.97)	12.21 (10.8113.50)	11.07 (10.8311.15)	13.20 (13.05-13.49)	2.61	6%	0.0016
Neurotrophin3§	3.24 (2.30-4.92)	3.04 (2.30-4.04)	2.30 (2.30-2.31)	3.97 (3.90-4.04)	2.30	6%	0.0018
C-X-C motif chemokine 19	10.38 (5.01-13.01)	9.79 (8.07-12.52)	8.55 (8.10-8.67)	12.22 (10.85-12.52)	2.40	8%	0.0019
Programmed cell death 1 ligand 1	6.52 (5.65-7.63)	6.39 (5.47-8.64)	5.41 (5.47-5.57)	7.60 (7.06-8.63)	2.73	9%	0.0035
Fms-related tyrosine kinase 3 ligand	10.47 (9.24-11.83)	10.28 (8.40-11.61)	9.01 (8.43-9.21)	11.20 (11.03-11.58)	2.58	6%	0.0054
TNF-related apoptosisinducing ligand	8.81 (7.36-9.67)	8.59 (7.47-9.34)	7.74 (7.48-7.93)	9.16 (9.10-9.34)	1.58	5%	0.0079
CD40L receptor	12.61 (11.59-14.29)	12.47 (11.4013.88)	11.61 (11.4111.71)	13.27 (13.1013-85)	2.83	5%	0.0079
Interferon gamma	8.05 (6.43-10.09)	7.68 (6.28-10.41)	6.50 (6.29-6.67)	10.25 (9.67-10.41)	4.71	7%	0.0081
TNF-related activationinduced cytokine	5.84 (3.51-7.34)	5.59 (3.39-7.18)	4.30 (3.39-4.46)	6.69 (6.49-7.14)	1.82	7%	0.0090
Urokinase-type plasminogen activator	10.61 (9.45-11.86)	10.41 (9.19-11.50)	9.40 (9.19-9.69)	11.41 (11.08-11.50)	2.13	5%	0.0110
C-X-C motif chemokine 1	10.88 (9.54-12.35)	10.67 (9.73-12.59)	9.73 (9.17-9.96)	11.83 (11.50-12.59)	3.81	6%	0.0152

Fibroblast growth factor 21	4.79 (2.56-8.37)	4.45 (2.56-8.34)	2.56 (2.56-2.78)	7.50 (6.56-8.33)	2.56	8%	0.0166
Fractalikine	5.45 (4.50-6.71)	5.35 (3.81-6.73)	4.17 (3.82-4.53)	6.20 (6.14-6.70)	2.71	7%	0.0273
Osteoprotegerin §	11.01 (10.16-12.28)	10.92 (9.44-11.80)	9.78 (9.44-10.08)	11.64 (11.51-11.80)	0.24	6%	0.0274
T cell surface glycoprotein CD6 isoform	7.57 (6.09-8.74)	7.40 (5.92-8.32)	6.07 (5.93.6.48)	8.27 (8.11-8.32)	2.70	6%	0.0274
Tumour necrosis factor ligand superfamily member 14	8.00 (6.00-10.03)	7.73 (5.98-9.51)	6.25 (6.00-6.73)	8.96 (8.86-9.49)	2.56	6%	0.0323
Leukaemia inhibitory factor	1.06 (1.06-2.44)	1.06 (1.06-2.34)	1.06 (1.06-1.06)	1.38 (1.12-2.34)	2.11	7%	0.0355
Adenosine Deaminase	6.46 (4.02-8.04)	6.25 (5.15-7.52)	5.38 (5.15-5.62)	7.18 (7.02-7.50)	1.73	5%	0.0355
TNF-beta	6.14 (4.77-7.16)	5.88 (4.50-7.34)	4.84 (4.51-5.12)	6.92 (6.83-7.34)	2.12	6%	0.0417
**Decreased**							
Protein S100A12	6.35 (3.93-9.30)	7.27 (4.39-9.28)	4.92 (4.40-5.55)	9.08 (8.90-9.27)	2.13	8%	0.0000
Sulfotransferase 1A1	4.01 (2.25-6.48)	4.76 (2.55-6.58)	3.11 (2.57-3.41)	6.47 (5.97-6.58	2.25	6%	0.0000
Axin-1	2.77 (1.92-5.66)	3.17 (2.16-4.48)	2.31 (2.16-2.49)	4.27 (4.02-4.67)	1.92	6%	0.0000
Interleukin-17C	2.71 (1.84-5.26)	3.15 (2.03-7.63)	2.21 (2.03-2.34)	4.73 (4.23-7.61)	1.84	8%	0.0002
Caspase-8	4.93 (3.58-9.69)	5.35 (4.20-7.71)	4.50 (4.22-4.64)	6.51 (6.40-7.70)	2.63	7%	0.0006
STAM-binding protein	4.81 (3.82-7.16)	5.07 (3.91-8.20)	4.13 (3.91-4.40)	7.09 (6.35-8.18)	1.99	5%	0.0018
Fibroblast growth factor 19	9.58 (7.55-12.22)	9.96 (6.96-12.32)	12.08 (11.7112.32)	7.65 (6.96-8.17)	7.63	6%	0.0441

SIR2-like protein 2	3.66 (2.70-6.71)	3.97 (2.70-7.97)	2.85 (2.70-3.08)	6.49 (5.67-7.96)	7.63	8%	0.0053
Eukaryotic translation initiation factor 4E-binding protein 1[§]	6.65 (3.44-10.44)	7.13 (3.95-11.60)	4.40 (3.95-5.12)	10.91 (10.11-11.60)	2.08	6%	0.0064
Matrix metalloproteinase-1[‡,§]	16.04 (13.49-17.03)	16.27 (14.0417.22)	14.56 (14.0414.87)	17.0 (16.90-17.22)	1.91	5%	0.0098

GD, Graves` disease; CI, confidence interval; LOD, lower limit of detection; CV, coefficient of variation.

[*]Benjamin Hochberg procedure was applied to adjust p-values for statistical significance to control for false discovery rate due to multiple comparison.

[†]One outlier was excluded from analyses defining normal distribution.

[‡]Serum level of biomarker correlates with s-Thyroxine.

[§]Serum level of biomarker correlates with s-Thyrotropin receptor antibody.
